# Titanium Nitride as an Intermetallic Diffusion Barrier for Hydrogen Permeation in Palladium–Vanadium Composite Membranes

**DOI:** 10.3390/membranes15030068

**Published:** 2025-02-21

**Authors:** Cameron M. Burst, Chao Li, Douglas Way, Colin A. Wolden

**Affiliations:** 1 Materials Science Program, Colorado School of Mines, Golden, CO 80401, USA; burst@mines.edu; 2 Department of Chemical and Biological Engineering, Colorado School of Mines, Golden, CO 80401, USA; chaoli@mines.edu (C.L.); dway@mines.edu (D.W.)

**Keywords:** hydrogen membranes, titanium nitride, palladium, vanadium, intermetallic diffusion, diffusion barrier

## Abstract

Hydrogen purification is a critical industrial process, and there are ongoing efforts to develop low-cost alternatives to palladium foil membranes. Titanium nitride (TiN) is studied as an interdiffusion barrier to enable hydrogen permeation in composite palladium–vanadium membranes. TiN was deposited via reactive sputtering, and films with the desired (200) orientation were obtained in the metallic regime at 400 °C under a 200 V bias to the substrate. The permeability of thin-film TiN was determined with palladium-based sandwich structures. TiN layers up to 10 nm resulted in a minimal decrease in flux (~20%) relative to a freestanding PdCu foil, which was attributed to the interfacial resistance. At greater thicknesses, the TiN layer was rate-limiting, and it was found that the effective permeability of the sputtered TiN thin films was ~6 × 10^−12^ mol s^−1^ m^−1^ Pa^−0.5^. Composite Pd|TiN|V|TiN|Pd membranes exhibited permeability values up to three times greater than pure palladium, exhibiting stability at 450 °C for over 100 h, with the lack of intermetallic diffusion and alloy formation being confirmed with XRD. The membranes were unstable at 500 °C, which was attributed to the instability of the thin Pd layer and loss of catalytic activity.

## 1. Introduction

Common hydrogen purification methods such as cryogenic distillation and pressure swing adsorption require high amounts of energy. Dense metal membranes provide an economic, low-energy alternative [1]. Palladium is the standard material for these membranes, offering high flux and perfect selectivity. However, due to its rarity and the subsequent cost of this material [2], there are strong incentives to minimize the amount of palladium that is used in these membranes. Metals with a body-centered cubic (BCC) crystal structure such as Ta, Nb, and V are cheaper alternatives with higher bulk permeability than palladium. Unfortunately, these materials have a high affinity for oxygen and other contaminants, which rapidly degrade their ability to catalyze the dissociation and recombination of hydrogen [3].

To overcome these kinetic limitations, a thin catalyst layer is applied. Pd coatings create composite membranes that are capable of reaching theoretical permeabilities but rapidly degrade due to the intermetallic diffusion that is accelerated by hydrogen permeation [4]. There are two approaches to overcome this. The first is to apply non-metallic catalysts such as transition metal carbides including molybdenum (Mo_2_C) [5], titanium carbide (TiC) [3], vanadium carbide (VC) [6], and niobium carbide (NbC) [7]. While these materials were initially successful catalyst layers, in long-term studies, it was shown that the compound degraded and that carbon dissolved into the underlying vanadium [8]. The search for an active catalyst that has long-term stability, particularly in reactive environments, remains elusive. An alternative approach is to use a thin layer of Pd catalysts. This requires the presence of another layer that allows for hydrogen diffusion while preventing intermetallic diffusion between the base metal and Pd. Oxides are commonly used intermetallic diffusion barriers [9,10], but they will form an oxide with BCC metals that inhibits hydrogen permeation. A thermodynamic analysis (Ellingham diagrams) indicated that group IV metal nitrides (ZrN, TiN, HfN) should be stable with respect to BCC metals [11]. Indeed, HfN has been used for this purpose in composite Pd|HfN|Ta membranes [12,13]. This layer was effective at enhancing stability but significantly attenuated the permeability. More recently, we demonstrated that ZrN was also effective and achieved permeabilities that were up to three times greater than pure Pd in symmetric Pd|ZrN|V|ZrN|Pd membranes [14]. These membranes displayed excellent stability at T ≤ 450 °C (>200 hrs) but failed when the temperature was elevated above this value. This was expected from thermodynamics, as this transition coincides with the approximate temperature when ZrN will start to decompose to Z_r_N_y_ + N_2_ [15].

In this paper, we explore the potential of titanium nitride (TiN) to serve as a hydrogen-permeable, intermetallic diffusion barrier. TiN offers much better thermal stability, with a nominal decomposition temperature of ~2900 °C, and finds practical use as a thin film at temperatures up to 1000 °C [13]. TiN may be considered an odd choice, since it has been pursued as a hydrogen permeation barrier due to its excellent adhesion and ease of deposition in addition to thermal stability [16,17]. While TiN can be an excellent hydrogen barrier, its hydrogen transport properties depend strongly on its morphology and crystal orientation, with its reported barrier performance varying by several orders of magnitude [17]. For example, Zhou et al. [18] showed that TiN films with a (111) texture were better hydrogen barriers than films with a (200) texture. Moreover, Kura et al. [19] achieved significant hydrogen permeance in nanocrystalline TiN membranes with sub-stoichiometric compositions (TiN_x_, 0.7 < x < 0.95). The impressive H_2_ permeability was attributed to surface diffusion and improved with a reduced grain size.

In this study, it was shown that TiN can serve as a hydrogen-permeable, intermetallic diffusion barrier to enable high-performance composite vanadium membranes for high-temperature hydrogen purification. TiN was deposited by reactive sputtering and characterized with X-ray diffraction, four-point probe resistivity, and profilometery. The temperature, composition, and substrate bias were used to control the film orientation. The permeability of the TiN thin films was assessed using Pd sandwich structures. It was found that TiN films in the 5–10 nm thickness range were effective intermetallic diffusion barriers while still retaining significant hydrogen permeation. When employed in Pd|TiN|V|TiN|Pd composite membranes, a performance exceeding that of pure Pd was achieved. However, the thermal stability was not improved. Intermetallic diffusion was not observed, so the loss in performance is attributed to the thermal instability of the thin (100 nm) Pd catalyst layers, whose structure degraded when heated above 450 °C.

## 2. Materials and Methods

### 2.1. Fabrication

TiN was fabricated via magnetron sputtering with an ATC Orion 5 sputter system (AJA International, Hingham, MA, USA). Silicon wafers, glass slides, vanadium foils (ESPI, 100 µm), and Pd_60_Cu_40_ foils (ATI Specialty Alloys & Components, 25 µm) were employed. The first two were used as witness samples for the subsequent characterization of film thickness, electric resistivity, and crystal structure. Before deposition, these samples were cleaned in an Ar plasma at 5 mTorr with 50 W RF reverse bias for 30 min to remove surface contamination. Without re-exposure to air, TiN was deposited in an N_2_/Ar ambient using DC sputtering from a 2” diameter Ti target. The total N_2_/Ar flow rate and operating pressure were fixed at 15 sccm and 5 mTorr, respectively. TiN deposition was studied as functions of the substrate temperature, substrate bias, nitrogen concentration, and film thickness. The TiN was tuned to deposit at one nm/min, with a set power of 65 W, resulting in 354 V and 183 mA across the target. Pd thin films were also deposited by DC sputtering with 165 mA of current applied to a 2” Pd target in a 5 mTorr Ar ambient. The resulting Pd deposition rate was ~6.67 nm/min, and the Pd thickness was fixed at 100 nm. For the fabrication of composite membranes, one side was completed without breaking the vacuum before removing the sample from the chamber and flipping it and then performing the same cleaning and deposition procedures again.

### 2.2. Characterization and Testing

TiN thin films were simultaneously deposited on V foils and Si and glass witness samples. The silicon substrates were used for thickness and XRD measurements, while the glass was used for electrical resistivity. The crystal structure of the TiN films was examined with a PANalytical X-ray Diffractometer with Cu K*α* radiation at 45 kV in the configuration of 2-scan. A KLA Tencor-D600 profilometer was used to determine the deposition rate by measuring the step size of the deposited films. A linear Ossila four-point probe was used to measure the sheet resistance of the TiN films.

Hydrogen permeation measurements were conducted by sealing symmetric (Pd|TiN|V|TiN|Pd) and asymmetric (PdCu|TiN|Pd) membranes into a commercial ½” Swagelok VCR^®^ cell (Solon, Ohio, USA), which resulted in an effective surface area of 0.93 cm^2^. The asymmetric membranes were mounted so that the 100 nm layer of Pd faced the feed side (high pressure), while the bare PdCu faced the permeate side (low pressure). The membranes were heated under inert gas UHP He or N_2_ flow (General Air, Denver, CO, USA) in a Lindberg Blue tube furnace at a rate of 3.33 °C/min to the desired temperatures (400–600 °C). Once the membrane reached the operating temperature, the membrane was exposed to 50 sccm of H_2_ on the feed side and 20 sccm on the permeate side for at least three minutes, while the feed side pressure increased to 50 psig. The permeate side remained at or near ambient pressure (83 kPa in Golden, CO, USA). The membranes were periodically exposed to UHP He to ensure that there were no leaks and confirm the infinite H_2_ selectivity of these membranes.

The permeability (π) can be determined using the measured H_2_ flux (J), the partial pressures on the feed and permeate side (P_f_ and P_p_, respectively), and the thickness of the foil (L) using Sieverts’ Law:

(1) $$\pi =\frac{L\times J}{P_{f}^{0.5}-P_{p}^{0.5}}$$

The use of *n* = 0.5 assumes that permeation is limited by the bulk diffusion, which is justified through the application of Pd, which provides rapid reactions at the surfaces.

## 3. Results

### 3.1. TiN Properties

**Figure 1:**
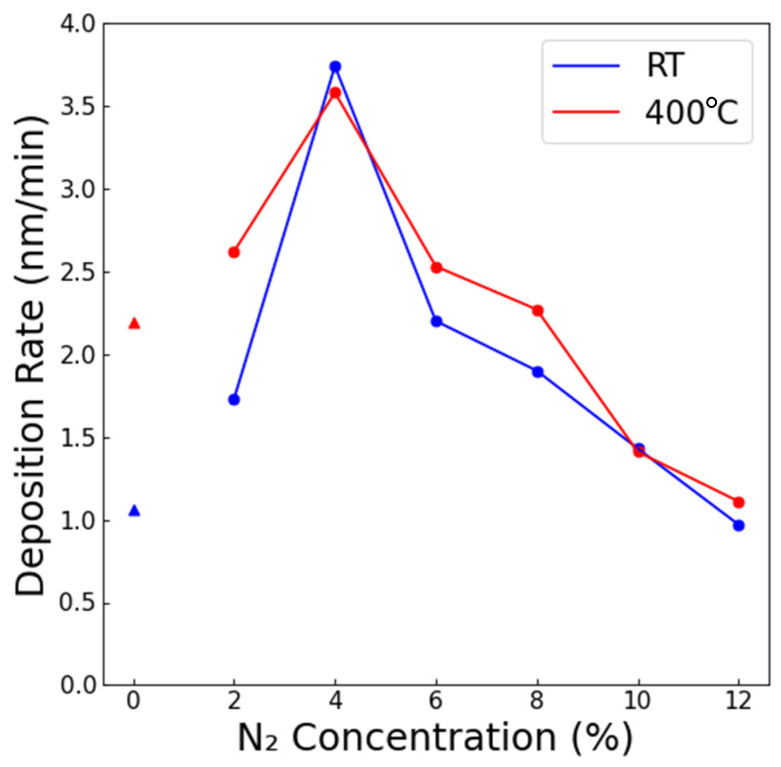
The measured deposition rate of TiN as a function of the sputter ambient concentration at both room temperature (blue) and 400 °C (red). Samples deposited at 0% N_2_ are metallic titanium and indicated with triangles to distinguish from TiN (circles).

TiN was deposited via reactive sputtering. Figure 1 plots the deposition rate as a function of the gas composition (% N_2_) at both room temperature and at T = 400 °C substrate temperatures. Note that the films that were deposited without N_2_ present were of course titanium metal and are included for reference. Upon nitrogen addition, the rate initially increased, with a maximum at 4% N_2_, and subsequently declined at higher N_2_ concentrations. Reactive sputtering is classified by three modes of operation: the metallic, transition, and compound regimes [20,21]. At low reactive gas concentrations, the target surface remained metallic, and the deposition rates were high. At high reactive gas concentrations, the deposition rates were low and stable, as the target surface was coated with the ceramic, which is more difficult to sputter. A transition regime existed between these two extremes. The range examined in this work spanned from the metallic mode through the transition regime. The relatively low deposition rates at 2% suggest that stoichiometric TiN was not being formed, as the rates were relatively similar to those of metallic Ti. The highest deposition rate was observed at 4% N_2_, and the rates declined as the N_2_ concentration increased, which is characteristic of the transition regime. Approaching 12% N_2_, the rates appeared to start leveling off, characteristic of the compound regime.

**Figure 2:**
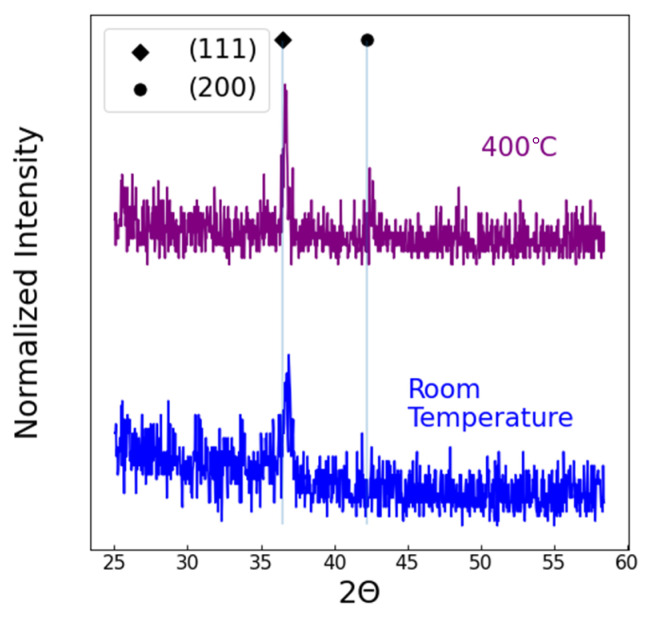
The XRD pattern of 100 nm thick TiN deposited on silicon at room temperature and at 400 °C with no applied bias.

As expected, the substrate temperature had little impact on the deposition rate, but the films that were deposited at 400 °C displayed better crystal orientation. Figure 2 compares the XRD patterns of the films that were deposited at room temperature and at 400 °C using 4% N_2_. As discussed above, the (111) crystal orientation is a better hydrogen barrier, and (200) is a better permeator. At room temperature, the TiN was preferentially in the (111) orientation. At T = 400 °C, the desired (200) orientation appeared in the XRD patterns.

**Figure 3:**
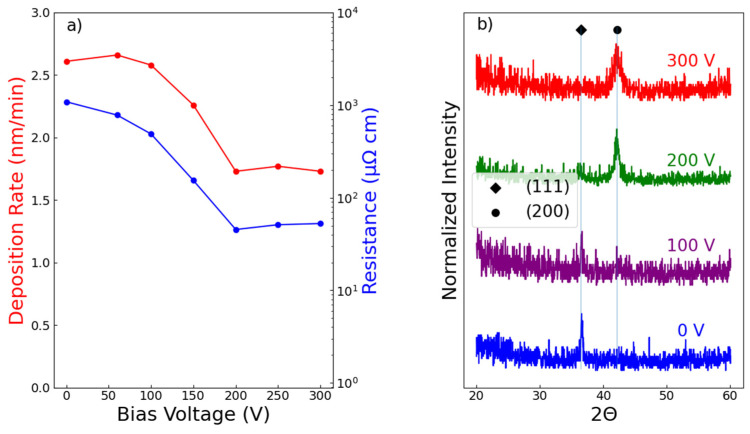
(**a**) The measured deposition rate (red, left axis) and resistivity (blue, right axis) of 100 nm TiN films on glass as a function of applied reverse RF bias. (**b**) XRD patterns of 100 nm TiN films on silicon as a function of applied bias.

It is well known that bias can have a significant impact on the quality and morphology of sputtered TiN films [18,21,22,23]. Figure 3a plots the impact of bias on both the deposition rate and electrical resistivity. As shown, the two quantities track each other, both decreasing with the applied voltage before saturating at 200 V. Resistivity is indicative of film composition, with low values indicating high purity. Our findings are consistent with a previous study [24] that also achieved low-resistance and high-purity TiN with a combination of 200 V bias and substrate heating. Bias also has a profound effect on the crystal structure (Figure 3b). Films that were deposited without a bias displayed a preferential (111) orientation. This signal was attenuated by increasing the bias to 200 V or greater, and the films were textured in the desired (200) orientation. Based on these initial studies, TiN films that were deposited with a combination of 4% N_2_, Ts = 400 °C, and 200 V bias were employed for use as intermetallic diffusion barriers. These films exhibited low resistivity and the desired orientation and were expected to be thermally stable, since the deposition temperature was similar to conditions used for hydrogen permeation.

### 3.2. Composite Membrane Properties

The common approach to measuring the permeability of hydrogen barriers is through transient pressure curves based on the lag time and steady state permeation [18,25]. These experiments are long and require the use of a very high-quality vacuum systems [26], which is challenging for the very thin films of interest. It can also be difficult to decouple the contributions of surface kinetics and bulk permeability. To overcome these challenges, we assessed the hydrogen permeability of the TiN thin films by depositing them on PdCu foils and then overlaying 100 nm of Pd. The use of this Pd-based sandwich structure eliminates surface limitations, and the resistance of the TiN layer is determined by the permeance with the base PdCu foil.

**Figure 4:**
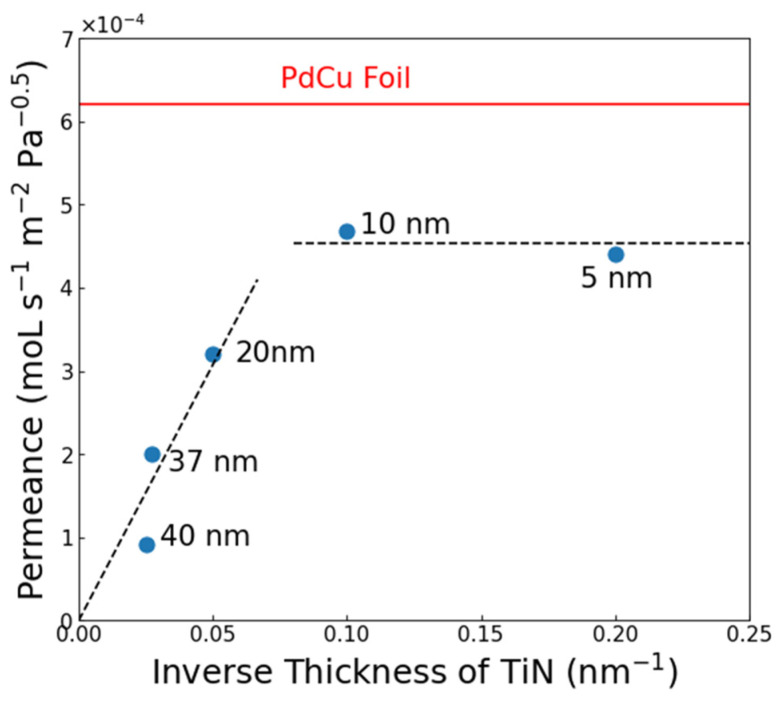
H_2_ permeance versus inverse TiN thickness for Pd|TiN|PdCu membranes at 400 °C. The value of an uncoated PdCu foil is included for reference.

Figure 4 compares the permeance of TiN films of varying thicknesses in the PdCu sandwich structure. Testing was carried out at 400 °C with a pressure gradient of ∆P = 50 psi. For 5 and 10 nm TiN films, the permeance was reduced by ~20% relative to the value that was obtained from the freestanding PdCu foil, which is attributed to the interfacial resistance introduced by the TiN layer. As the thickness of TiN was increased above 10 nm, the permeance dropped roughly linearly with respect to the inverse thickness, which is indicative that transport through TiN is rate-limiting. From the slope shown in Figure 4, the permeability of the sputtered TiN is estimated to be ~6 × 10^−12^ mol s^−1^ m^−1^ Pa^−0.5^. This value is comparable to the permeability that was obtained by Kura and co-workers [19] for TiN membranes that were prepared by reactive sputtering with thicknesses ranging from 200 to 2500 nm (~10^−11^ mol s^−1^ m^−1^ Pa^−0.5^ at T = 400 °C).

**Figure 5:**
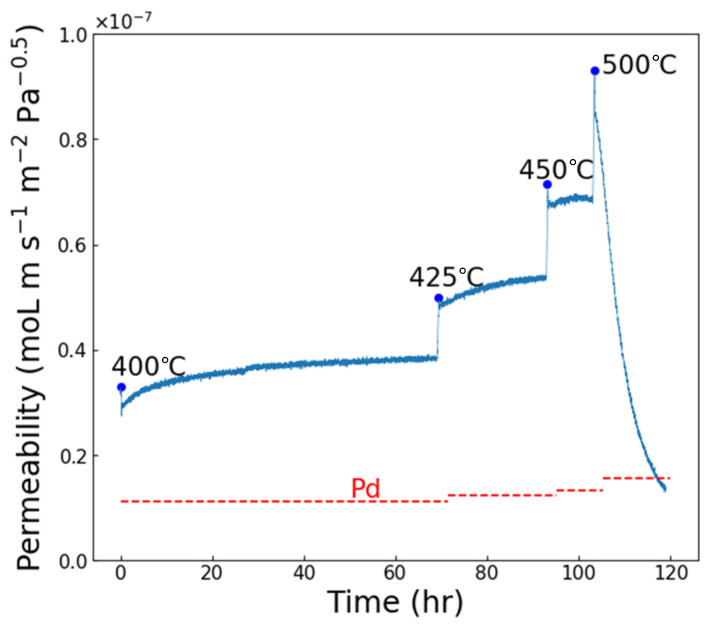
The H_2_ permeability through a 5 nm Pd|TiN|V|TiN|Pd composite membrane as a function of time at selected temperatures. The horizontal lines indicate the theoretical permeability of pure Pd at the selected temperature.

Based on this testing, TiN layers of 5 and 10 nm were used to form composite Pd|TiN|V|TiN|Pd membranes for hydrogen permeability testing. Figure 5 shows a permeation test of a composite membrane with 5 nm thick TiN. Testing commenced at T = 400 °C, with an initial permeability of ~3 × 10^−8^ mol m^−1^ Pa^−0.5^ s^−1^, which slowly increased and stabilized over the course of 65 h. Stepping up the temperature to 425 and 450 °C brought additional improvements while maintaining stability. The permeability jumped when the temperature was further increased to 500 °C but then rapidly failed.

**Figure 6:**
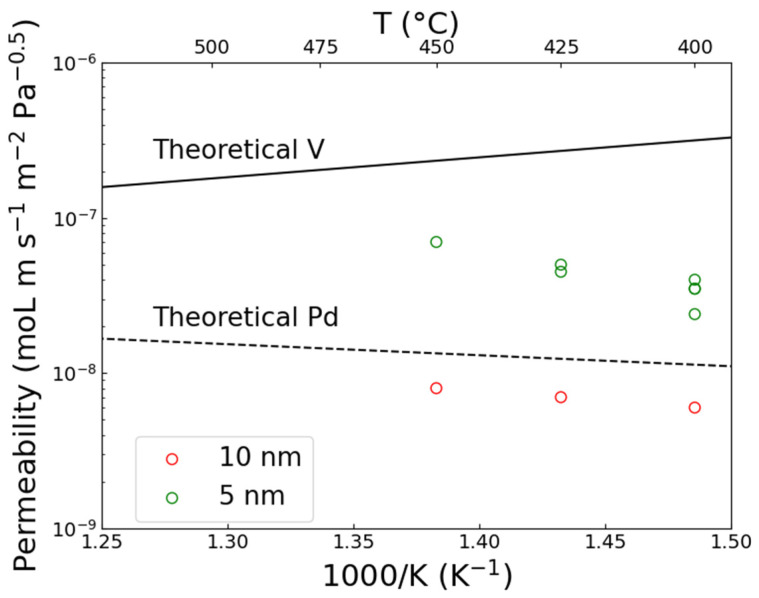
Arrhenius plot comparing the permeability of Pd|TiN|V|TiN|Pd membranes with values of Pd and V from the literature.

Figure 6 displays an Arrhenius plot comparing the performance of composite Pd|TiN|V|TiN|Pd membranes with the theoretical values of V and Pd. For composites with 5 nm TiN, the permeability was roughly ~3x greater than that of Pd and between 2 and 8x less than V. The gap decreased as the temperature was raised due to their opposite temperature dependencies. However, when the TiN thickness was increased to 10 nm, the permeability was high but ~20% lower than that of pure Pd. This contrasts with the Pd sandwich structures (Figure 4), where 5 and 10 nm had identical performances. This suggests that the barrier at the TiN|V interface is greater than that in the TiN|PdCu interface.

## 4. Discussion

The results obtained here were very similar to those for composite membranes fabricated with Mo_2_C [5] and ZrN [14] interdiffusion barriers. With respect to other vanadium composite membranes, these Pd-based composites outperformed vanadium coupled with carbide catalysts [3, 5–7] at temperatures of 400–450 °C, but vanadium composites employing carbide catalysts displayed greater performance and better stability at elevated temperatures. Notably, vanadium composites employing vanadium carbide [6] and niobium carbide [7] catalysts have achieved hydrogen permeabilities approaching that of pure vanadium at T = 700 °C.

**Figure 7:**
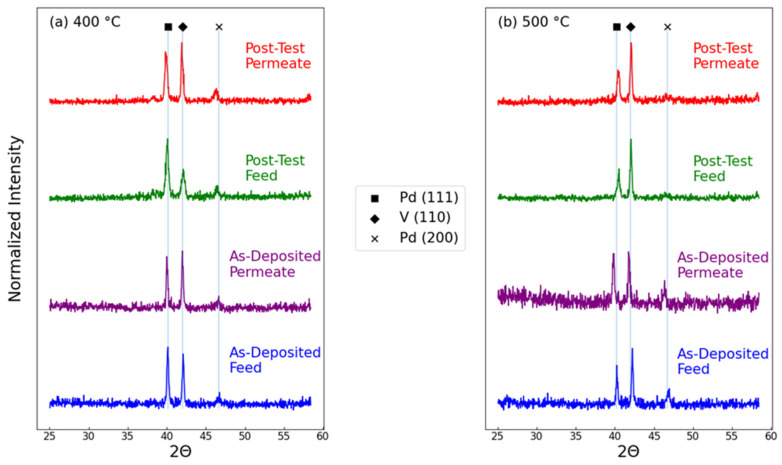
XRD patterns of composite membranes before and after H_2_ permeation testing at (**a**) 400 °C and (**b**) 500 °C.

To assess the reasons for the instability at T = 500 °C, an XRD analysis was performed on the membranes before and after testing, as shown in Figure 7. In these membranes, the only signals that were detected arose from the V foil and the 100 nm Pd layer. The thin TiN layer was below the detection limits. As expected, the XRD patterns were nominally identical among the samples before testing on both the feed and permeate side. After long permeation testing at T = 400 °C, the XRD pattern was nominally unchanged. When intermetallic diffusion occurred, shifts were observed in the position of both the V and Pd peaks due to alloy formation [4]. After testing at T = 500 °C, the positions of both V and Pd remain unchanged, suggesting negligible diffusion and alloy formation. However, while the intensity of the V signals remained unchanged, the Pd signal was degraded on both the feed and permeate side. The (200) nearly vanished, and the (111) signal was greatly attenuated. This suggests that at 500 °C, the Pd catalyst layer was unstable, with thermal migration leading to disorder and a loss of catalyst activity.

Over the years, we have studied composite membranes with different barrier layers between Pd and V, including Mo_2_C [5], ZrN [14], and now TiN. In every case, the temperature stability limit was in the range of 450–500 °C. This suggests that the primary factor is not the interlayer, but the stability of the Pd catalyst layer. While sufficient for lower temperature applications, this suggests that different catalysts are needed for applications operating at temperatures greater than 450 °C.

## 5. Conclusions

Composite Pd|TiN|V|TiN|Pd membranes were investigated for high-temperature H_2_ separation. The TiN layers were deposited by reactive sputtering, with the desired (200) orientation being achieved with a combination of bias and elevated temperature. The thin-film TiN’s permeability was assessed using Pd-based sandwich structures, yielding values that were comparable to what was observed in much thicker (200–2500 nm) TiN membranes. TiN layers up to 10 nm thick had a negligible impact on permeance. The hydrogen permeabilities of the composite membranes were up to 3x greater than those of pure Pd and stable up to 450 °C. Increasing the temperature to 500 °C resulted in rapid degradation. Post-mortem testing did not reveal evidence of intermetallic diffusion or alloy formation, but rather degradation of the Pd catalyst layer due to thermal migration. It is suggested that 450 °C is the limit for composite membranes employing Pd, regardless of the interlayer, and alternative catalysts are required to enable operation at elevated temperatures.

## Data Availability

The original contributions presented in this study are included in the article/Supplementary Materials. Further inquiries can be directed to the corresponding author(s).

## Supplementary Materials

The following supporting information can be downloaded at https://www.mdpi.com/article/10.3390/membranes15030068/s1: An Excel file containing the raw data.

## Abbreviations

The following abbreviations are used in this manuscript:

TiN	Titanium nitride
Pd	Palladium
PdCu	Palladium copper
V	Vanadium
ZrN	Zirconium Nitride
XRD	X-ray diffraction
